# Inhibition of Purine Metabolism Promotes the Differentiation of Neuroblastoma Driven by *MYCN*

**DOI:** 10.1002/cam4.70953

**Published:** 2025-05-12

**Authors:** Yufeng Jiang, Hui Xiao, Yi Yang, Guoyu Chen, Yingwen Zhang, Xiaoyu Wu, Qi Zhang, Qingyi Huang, Hongxiang Gao, Yali Han, Anan Zhang, Dapeng Jiang, Liang Zheng, Yanxin Li, Yijin Gao

**Affiliations:** ^1^ Department of Oncology, Shanghai Children's Medical Center, Shanghai Jiao Tong University School of Medicine Key Laboratory of Pediatric Hematology & Oncology of China Ministry of Health Shanghai China; ^2^ Pediatric Translational Medicine Institute, Shanghai Children's Medical Center, Shanghai Jiao Tong University School of Medicine Key Laboratory of Pediatric Hematology & Oncology of China Ministry of Health Shanghai China; ^3^ State Key Laboratory of Oncogenes and Related Genes, Renji‐Med X Clinical Stem Cell Research Center, Renji Hospital, Shanghai Cancer Institute Shanghai Jiao Tong University School of Medicine Shanghai China; ^4^ Department of General Surgery, Shanghai Children's Medical Center Shanghai Jiao Tong University School of Medicine Shanghai China

**Keywords:** lometrexol, neuroblastoma, purine metabolism, tumor differentiation

## Abstract

**Background:**

Neuroblastoma (NB), the common extracranial solid tumor in children, is associated with a poor prognosis, particularly in high‐risk patients. *MYCN* amplification stands as the most prominent molecular hallmark within this high‐risk subgroup. However, MYCN protein is considered “undruggable” due to its lack of a conventional enzymatic binding pocket and its predominant nuclear localization, which precludes targeting by standard small‐molecule inhibitors or antibody‐based therapeutics. Consequently, current therapeutic strategies have achieved limited efficacy against MYCN‐driven NB. Notably, MYCN not only orchestrates diverse metabolic reprogramming pathways in tumors but also exerts a pivotal influence on cellular differentiation. To overcome this therapeutic barrier, we seek to elucidate the contribution of purine metabolism to stemness maintenance in *MYCN*‐amplified NBs and to discover novel small‐molecule inhibitors capable of inducing differentiation in high‐risk NBs.

**Methods:**

Metabolomic profiling via mass spectrometry was employed to delineate differential metabolite signatures between *MYCN*‐amplified and non‐amplified NB cells. Bioinformatics analysis of publicly available RNA sequencing datasets facilitated the systematic evaluation of purine metabolic enzyme expression. Cell differentiation, proliferation, colony formation, and cell migration assays were employed to assess the inhibitor's effects. Additionally, an in vivo xenograft model of NB was established to examine the therapeutic potential of lometrexol (LMX), a selective inhibitor of the purine biosynthesis enzyme phosphoribosylglycinamide formyltransferase (GART).

**Results:**

Significant changes in nucleotide metabolism were identified in NB cell lines with high *MYCN* expression compared to those with low *MYCN* expression. The expression of purine metabolic enzyme genes was positively correlated with *MYCN* expression, prognosis, and differentiation status in NBs. Pharmacological inhibition of GART using LMX elicited a robust pro‐differentiation response, concomitant with a significant suppression of tumorigenic potential.

**Conclusions:**

These findings establish purine metabolic enzyme inhibition as a viable therapeutic strategy to induce differentiation and attenuate tumor progression in high‐risk *MYCN*‐amplified NBs.

**Abbreviations**
ADSL: adenylosuccinate lyase
AICAR: 5‐aminoimidazole‐4‐carboxamide1‐β‐D‐ribofuranoside
ATIC: 5‐aminoimidazole‐4‐carboxamide ribonucleotide
EFS: event‐free survival
FGAM: 2‐(formamido)‐N1‐(5′‐phosphoribosyl) acetamidine
GART: phosphoribosylglycinamide formyltransferase
GMP: guanosine monophosphate
GMPS: guanine monophosphate synthase
GNB: ganglioneuroblastoma
H&E: hematoxylin–eosin
HR: hazard ratio
IHC: immunohistochemistry
IMP: inosinemonophosphate
IMPDH1: inosine monophosphate dehydrogenase 1
ISL1: insulin gene enhancer binding protein 1
KEGG: Kyoto Encyclopedia of Genes and Genomes
LMX: lometrexol
MAP 2: microtubule‐associated protein 2
MKI: mitotic–karyorrhexis index
MTHFD2: methylenetetrahydrofolate dehydrogenase 2
*MYCN*‐Amp: *MYCN*‐amplification
*MYCN*‐Non: *MYCN*‐non‐amplification
NB: neuroblastoma
NDRG1: N‐Myc downstream regulated 1
NeuN: neuronal nuclear antigen
OCT4 or POU5F1: POU class 5 homeobox 1
OS: overall survival
PAICS: phosphoribosylaminoimidazolesuccinocarboxamide synthase
PFAS: phosphoribosylformylglycinamidine synthase
PPAT: phosphoribosyl pyrophosphate amidotransferase
PRPH: peripherin
PRPS1: phosphoribosyl pyrophosphate synthetase 1
qRT‐PCR: RNA extraction and quantitative real‐time PCR
RA: retinoic acid
SCG2: secretogranin II
SOX2: SRY‐box transcription factor 2
TH: tyrosine hydroxylase
TUBB3: tubulin beta 3

## 1 Introduction

Neuroblastoma (NB) is a pediatric nervous system tumor that typically originates from undifferentiated neural crest cells, with 90% of cases diagnosed before the age of 5 [1]. *MYCN* amplification, occurring in ~25% of NBs, serves as a genomic hallmark of high‐risk disease that portends aggressive clinical behavior and poor prognosis [2]. MYCN drives oncogenesis by orchestrating ventral migratory patterns during early development, promoting clonal expansion, and transcriptionally activating gene networks that regulate survival, metastasis, proliferation, pluripotency maintenance, self‐renewal capacity, and angiogenesis [3]. The intrinsic structural features of MYCN, including its lack of druggable binding pockets and nuclear localization, have rendered it refractory to conventional pharmacological inhibition [4]. Consequently, identifying and targeting MYCN‐dependent downstream effector pathways has emerged as a critical therapeutic strategy.

MYCN serves as a master regulator of NB differentiation while simultaneously orchestrating profound metabolic rewiring, including dysregulation of nucleotide metabolism, aerobic glycolysis, and amino acid metabolism [5, 6]. Of particular significance, MYCN dramatically enhances de novo purine biosynthesis through coordinated upregulation of rate‐limiting enzymes, including PRPS1, PPAT, PFAS, PAICS, ADSL, and IMPDH1 [7]. Chromatin immunoprecipitation sequencing (ChIP‐seq) analysis with anti‐MYCN antibodies has definitively established direct transcriptional regulation of key metabolic nodes, including PAICS and the mitochondrial folate enzyme MTHFD2 [8]. Notably, emerging evidence from pluripotent stem cell models indicates that PRPS1/2‐mediated purine biosynthesis serves as a critical metabolic switch governing the balance between stemness maintenance and differentiation [9], suggesting potential conserved mechanisms in MYCN‐driven NB. Current pharmacological strategies targeting purine metabolism focus primarily on phosphoribosylglycinamide formyltransferase (GART) inhibition, with several clinically relevant compounds under investigation: lometrexol (LMX), pelitrexol (AG 2037), LY254155, LY222306, and pemetrexed disodium hemipentahydrate (LY231514) [10]. Among these, LMX has demonstrated particularly robust anti‐tumor efficacy across multiple malignancies, including breast cancer [11], leukemia [12], and lung cancer [13] with ongoing phase II (NCT00033722) and phase I (NCT00024310) clinical trials. However, despite these advances, the specific impact of LMX on MYCN‐driven tumorigenesis remains completely unexplored.

In this study, metabolic profiling identified MYCN as a critical regulator of purine metabolic reprogramming in NB. Targeting GART with LMX induced neuronal differentiation in NB cells and significantly attenuated tumorigenic potential in vitro and in vivo. These findings establish targeting purine metabolism, particularly through GART inhibition, as a promising differentiation therapy for *MYCN*‐amplified high‐risk NB.

## 2 Materials and Methods

### 2.1 Cell Lines

SH‐SY5Y cells with or without *MYCN* sgRNA were cultured in Dulbecco's modified Eagle medium (#C11995500BT, Gibco, Waltham, MA, USA), supplemented with 10% fetal bovine serum (FBS) (#WS500T, Ausbian, China) and 1% penicillin–streptomycin (#C100C5, New Cell & Molecular Biotech, China). CHP‐134 cells with or without *MYCN* sgRNA were cultured in RPMI Medium 1640 basic (#C11875500CP, Gibco) supplemented with 10% FBS and 1% penicillin–streptomycin. All of the cell lines were grown at 37°C in a 5% CO_2_ environment.

### 2.2 Plasmid

The complementary sense and antisense oligonucleotides encoding sgRNAs targeting *MYCN* were synthesized, annealed, and cloned into the lentiCRISPRv2 vector. The related sequences of sgRNAs: Crisper‐*MYCN* F: 5′‐caccgCGAGTGCGTGGATCCCGCCG‐3′, Crisper‐*MYCN* R: 5′‐aaacCGGCGGGATCCACGCACTCGc‐3′.

### 2.3 Cell Transfection and Lentivirus Infection

For transient transfection, cells were transfected using Hieff Trans Liposomal Transfection Reagent (Yeason). Crisper‐*MYCN* plasmid was cotransfected with packaging vectors psPAX2 and pMD2.G into HEK293T cells using Hieff Trans Liposomal Transfection Reagent (Yeason). Infectious lentivirus particles were harvested at 48 h after transfection, filtered through 0.45 μm PVDF filters, and then transduced into the NB cell line. At last, screen positive knockout clones by utilizing the method of limiting dilution.

### 2.4 Subcutaneous Xenograft Assays

Approval for all animal experiments was obtained from the Shanghai Jiao Tong University Institutional Animal Care and Use Committee (IACUC). The NOD/SCID mice were purchased from the Shanghai Model Organisms Center (Shanghai, China). NOD/SCID female mice, aged 6–8 weeks, were randomly grouped into three sets. To evaluate the impact of LMX, CHP‐134 cells (5 × 10^6^) were subcutaneously injected into the lower part of the abdomen of the mice. For the NB treatment assessment, intraperitoneal injections of either the vehicle or LMX (15 mg/kg) were administered every 3 days for two cycles, commencing when the tumors reached an average size of 200–250 mm^3^ considering animal ethics. Tumor volumes were monitored every other day. Upon completion, the mice were sacrificed with cervical dislocation, and the tumors were photographed.

### 2.5 RNA Extraction and Quantitative Real‐Time PCR (qRT‐PCR)

Total RNA from cell line extraction utilized AG RNAex Pro Reagent (#AG21101, Accurate Biology, China) following the manufacturer's guidelines. Reverse transcription follows the instructions for Hifair II 1st Strand cDNA Synthesis SuperMix for qRT‐PCR (#11123ES10, Yeasen Biotechnology, China). Cycling conditions were: 95°C for 5 min, then a total of 40 cycles of 95°C for 10 s, 60°C for 30 s, and 72°C for 1 min; followed by a final extension of 72°C for 10 min. qRT‐PCR was conducted on a CFX Connect Real‐Time PCR System (#1855201, Bio‐Rad Laboratories, Hercules, CA, USA) with ChamQ Universal SYBR qRT‐PCR Master Mix (#11201ES03, Vazyme, China). Normalization of target gene expression levels was performed relative to *Actin*. The primers are listed in Table S1.

### 2.6 Western Blotting

CHP‐134 cells (2 × 10^5^) were cultured in six‐well plates for 1 or 3 days. Cells were lysed in a buffer (20 mM Tris–HCl, pH 7.5, 150 mM NaCl, 1 mM EDTA, 2 mM Na_3_VO_4_, 5 mM NaF, 1% Triton X‐100, and protease inhibitor cocktail) at 4°C for 30 min as detailed previously [14]. Protein concentrations were measured, and equal amounts (10 μg) of protein from cell lysates were loaded onto 12% SDS‐PAGE gels. The membranes were incubated with anti‐MYCN [1:1000, #84406s, Cell Signaling Technology (CST), Danvers, MA, USA], anti‐β‐actin (1:1000, #3700T, CST), anti‐SOX2 (1:1000, #20118‐1‐AP, Proteintech, China), and anti‐NeuN (1:500, #269‐1‐AP, Proteintech) overnight at 4°C. Then the membranes were incubated with the secondary antibody anti‐HRP rabbit (1:3000, #7074, CST) or anti‐HRP mouse (1:3000, #7076, CST) at room temperature for 1 h. ECL reagents (#36208ES60, Yeasen Biotechnology) were used to develop protein bands.

### 2.7 Immunofluorescence Assay

Following fixation with 4% polyformaldehyde and permeabilization with 0.1% Triton X‐100 as detailed previously [15], cells underwent blocking with 10% goat serum in PBS. Subsequent staining involved TUBB3 (1:1000, #MMS‐435P, Biolegand, San Diego, CA, USA) and SCG2 (1:100, #20357‐1‐AP, Proteintech) antibodies. Visualization of the primary antibodies utilized goat anti‐mouse IgG bound to Alexa 647 (1:500, #ab150115, Abcam). DAPI was employed for nuclear staining, and fluorescence images were captured using a Zeiss inverted LSM confocal microscope (Carl Zeiss).

### 2.8 Hematoxylin–Eosin (H&E) Staining and Immunohistochemistry (IHC)

Sections from formalin‐fixed paraffin‐embedded samples underwent staining with H&E or IHC with anti‐Ki‐67 (1:100, #GT2094, Gene Tech, China), anti‐MYCN (1:200, #84406s, CST), or anti‐MAP2 (1:50, #4542, CST) antibodies following a standard immunohistochemistry protocol. Images were captured by confocal microscopy (STELLARIS 5, Leica) and the positive cells were counted.

### 2.9 Cell Growth Assay

Cells (2 × 10^4^) were seeded in 12‐well plates and cultured for 5 days, ensuring a minimum of three replicates per condition. Cell counts were performed daily using an automated cell counter from Bio‐Rad Laboratories. At approximately 80%–90% confluence, cells were digested by trypsinization and passaged.

### 2.10 Colony‐Forming Analysis

Cells were seeded at a density of 1500 per well in six‐well plates, ensuring a minimum of three replicates per condition. After 14–20 days, cells were fixed using 4% paraformaldehyde and stained with 0.05% crystal violet. Cell colonies were imaged using a microscope and counted.

### 2.11 Cell Apoptosis and Flow Cytometry Analysis

Cells were incubated in 12‐well plates with or without 70 ng/mL LMX for 24 h. The cells (1 × 10^5^) in each group were stained with the Annexin V‐APC and 7‐AAD (#640930, Biolegend), according to the manufacturer's recommendations, and analyzed by flow cytometry. Early apoptotic cells were marked as (Annexin V‐APC)^+^/(7‐AAD)^−^, and the late apoptosis was marked as (Annexin V‐APC)^+^/(7‐AAD)^+^. Our experiments included both early and late apoptosis. Flow cytometric data underwent a gating strategy to exclude cell debris and eliminate cell clusters, ensuring the acquisition of single cells. Each sample was processed with 1 × 10^5^ events. Data were collected using a BD FACSCanto flow cytometer (BD Biosciences). Subsequent analysis was performed using FlowJo V10 software.

### 2.12 Scratch Wound Healing Cell Migration Assay

Upon reaching 90% confluence in six‐well plates, cells were cultured in FBS‐free DMEM for 12 h. Subsequently, scratches were created using sterile 10 μL pipette tips. After three rinses with 1× PBS, cells were further cultured in DMEM without FBS, supplemented with LMX. Images of the scratches were captured at 0 and 24 h. Quantification of migrated cells to the wound area was performed by determining the relative percentage of the wound area covered by cells compared to the original wound area. This assay was repeated three times for analysis.

### 2.13 Metabolite Flux

Metabolite flux analysis by LC–MS was performed as described previously [9, 16]. Cells (1 × 10^6^) were centrifuged and washed twice. Methanol (80%) was added to resuspend the cell precipitate, and then the centrifuge tube was put into a 4°C oscillator at the highest speed to fully lyse the cells. After centrifugation at 21,130 × *g* for 10 min, the supernatant was removed for metabolite analysis by LC–MS. Significant differential metabolites were defined as |log2 (fold change)| ≥ 2 with *p*‐value < 0.05.

### 2.14 Bioinformatics Analysis

Metabolic pathway enrichment overview of differentially significant metabolites was employed using Metaboanalyst 5.0 and the KEGG database. Briefly, raw signals were pre‐processed, and the data were normalized. Univariate and multivariate statistical analyses were used to identify significantly expressed metabolites. MetaboAnalyst 5.0 (http://www.metaboanalyst.ca) online platform and KEGG database were used for metabolites pathway enrichment analysis.

### 2.15 Cell Viability Assay

Cell viability was assessed using the CellTiter‐Glo Luminescent Cell Viability Assay kit (#G7570, Promega, Madison, WI, USA) following the manufacturer's protocol. Cells were seeded in 96‐well plates at a density of 1 × 10^4^ cells per well and treated with serial concentrations of LMX for 72 h. Subsequently, 50 μL of CellTiter‐Glo reagent was added to each well and mixed for 10 min. Luminescence was then measured using a microplate reader (BioTek, Winooski, VT, USA). Concentrations of LMX were selected based on the measured IC_50_ values and relevant references.

### 2.16 Statistical Analysis

All data analyses were conducted with GraphPad Prism 9.0. Statistical comparisons were made using an unpaired Student's *t*‐test, one‐way ANOVA, or log‐rank (Mantel–Cox) test. A value of *p* < 0.05 was deemed statistically significant.

## 3 Results

### 3.1 MYCN Rewires NB Metabolism

**FIGURE 1:**
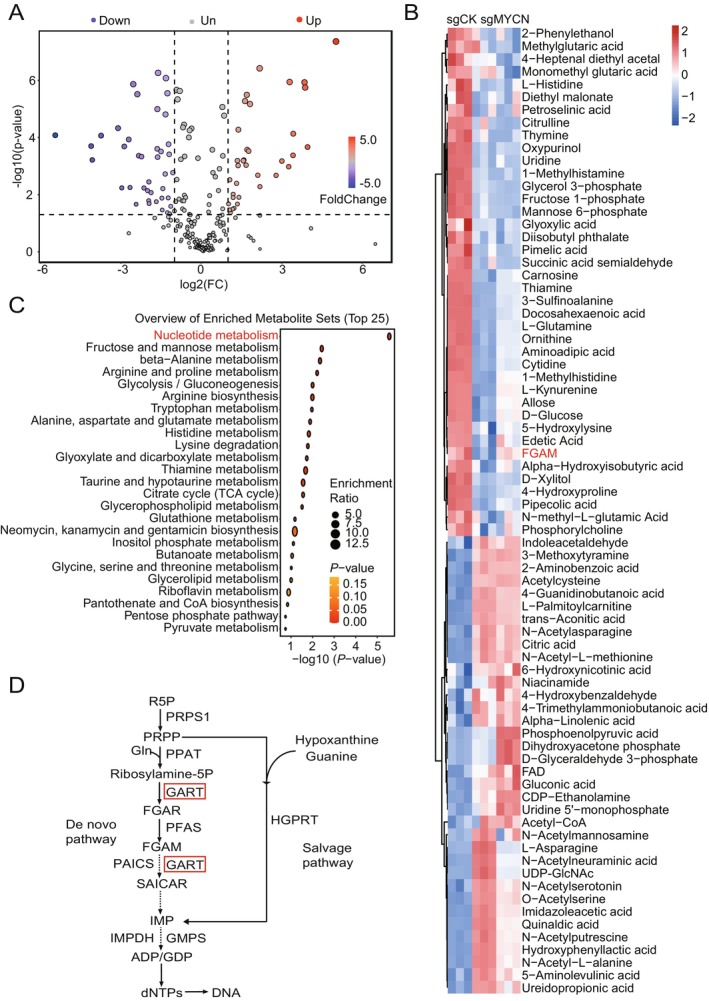
MYCN rewires NB metabolism. (A) Volcano plots of all metabolites by metabolic mass spectrometry to detect the effect of CHP‐134 cells with or without *MYCN* sgRNA on NB metabolism. sgCK is a control sgRNA. Significant differential metabolites were defined as |log2 (fold change)| ≥ 2 with *p*‐value < 0.05. (B) Heatmap of differentially significant metabolites in CHP‐134 cells with or without *MYCN* sgRNA. (C) Differential metabolites enrichment analysis of the KEGG obtained through Metaboanalyst 5.0 enrichment analysis. (D) Purine de novo synthesis and salvage pathway.

To systematically characterize MYCN‐mediated metabolic reprogramming, we performed untargeted mass spectrometry (MS)‐based metabolomic analysis in CHP‐134 NB cells with or without *MYCN* sgRNA. Volcano plot analysis identified 76 significantly dysregulated metabolites, comprising 36 upregulated and 40 downregulated species (Figure 1A). Unsupervised hierarchical clustering of these metabolites revealed clear segregation between *MYCN* sgRNA and the control groups, confirming the robustness of the metabolic alterations (Figure 1B, Figure S1A). Notably, we observed a marked reduction in FGAM, a key intermediate in de novo purine biosynthesis (Figure S1B). Kyoto Encyclopedia of Genes and Genomes (KEGG) pathway enrichment analysis further substantiated these findings, with nucleotide metabolism ranking among the top 25 most significantly perturbed pathways (Figure 1C). Purine nucleotide metabolism comprises two major pathways: the de novo synthesis pathway and the salvage pathway. Our data highlight three rate‐limiting enzymes—PRPS1 (PRPP synthetase), PPAT (glutamine amidotransferase), and GART (multifunctional trifunctional enzyme) as critical nodes in MYCN‐driven metabolic rewiring (Figure 1D). Of particular mechanistic significance, GART catalyzes three sequential reactions in the 10‐step de novo pathway [17]. Our integrated metabolomic and pathway analyses establish MYCN as a master regulator of de novo purine biosynthesis in NBs.

### 3.2 High Expression of Purine Nucleotide Metabolism Enzymes Predicts Poor Prognosis in NBs

**FIGURE 2:**
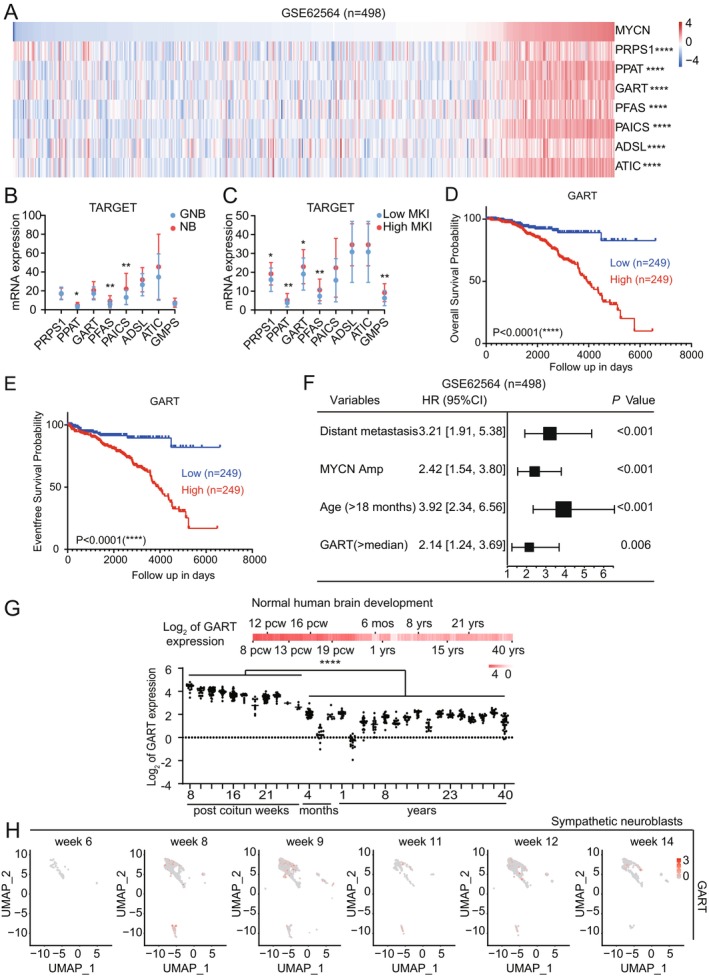
High expression of purine nucleotide metabolism enzymes predicts poor prognosis in NBs. (A) Spearman's correlation analysis of *MYCN* expression and purine metabolic enzyme gene expression in the GSE62564 database. (B, C) Purine metabolic enzyme gene expression in GN/GNBs and NBs (B), and low and high MKI subgroups (C) in the TARGET database. (D, E) Kaplan–Meier survival analysis of *GART* expression in the SEQC cohort (GSE62564). (D) Overall survival. (E) Event‐free survival. The best cut‐point was employed as the cutoff. (F) Multivariate Cox regression analysis of the prognostic ability of *GART* expression in the SEQC cohort (GSE62564). (G) Bar plot showing the log2 transformed fold‐change in *GART* expression during normal human brain development (upper panel) with grouped scatter plots (lower panel). pcw, post coitum weeks indicates post fertilization weeks. m, months. y, years indicates ages. Data from BrainSpan expression profiles (*n* = 524) in the R2 data platform (r2.amc.nl). (H) UMAP embedding showing the positions of sympathetic neuroblast cells with *GART* expression. *p < 0.05, ** *p* < 0.01, ****p < 0.0001, by unpaired two‐tailed *t*‐test (B, C), by log‐rank test (D, E).

To investigate the function of *MYCN* on purine nucleotide metabolism in NBs, we analyzed the RNA‐seq database GSE62564 and found the expression levels of the purine metabolic enzyme genes, *PRPS1*, *PPAT*, *GART*, *PFAS*, *PAICS*, *ADSL*, and 5‐aminoimidazole‐4‐carboxamide ribonucleotide (*ATIC*), positively correlated with *MYCN* expression (Figure 2A). We further revealed that *MYCN* KO in CHP‐134 cells reduced the expression of *PRPS1*, *PPAT*, *GART*, *PFAS*, *PAICS*, *ADSL*, *ATIC*, and guanine monophosphate synthase (*GMPS*) genes. In contrast, MYCN overexpression in SY5Y cells induced coordinated upregulation of *PRPS1*, *GART*, *PFAS*, *PAICS*, *ADSL*, *ATIC*, and *GMPS* genes (Figure S2A). These results establish *MYCN* as a critical regulator of purine nucleotide metabolism in NBs, mediated through transcriptional control of purine metabolic enzyme genes.

To delineate *MYCN*'s role in purine nucleotide metabolism, we performed integrative analysis of the NB RNA‐seq TARGET dataset. Strikingly, expression levels of key de novo purine biosynthesis enzymes were significantly elevated in NBs compared to the better‐differentiated ganglioneuroblastomas (GNBs) (Figure 2B). Notably, high mitotic‐karyorrhexis index (MKI) tumors, a known poor prognostic indicator, exhibited markedly increased expression of these genes (Figure 2C). Further stratification revealed consistent overexpression across multiple high‐risk categories, including *MYCN*‐amplified tumors, high‐risk group, progression group, and stage 4 group patients (Figure S2B–L). In addition, overall survival (OS) (Figure 2D) and event‐free survival (EFS) (Figure 2E) analyses indicated that high *GART* mRNA levels are related to a poor prognosis in NBs. EFS and OS analyses also revealed that high expression of purine metabolic enzyme genes, *PPRS1*, *PPAT*, *PFAS*, and *PAICS*, is related to a poor prognosis (Figure S3A,B). Multivariate analysis identified *GART* mRNA expression as an independent risk factor, comparable to established risk markers such as distant metastasis, *MYCN* amplification, and age at diagnosis (Figure 2F). Further multivariate analyses identified additional purine metabolic enzyme genes, *PPRS1*, *PPAT*, *PFAS*, and *PAICS*, as independent risk factors (Figure S4A–G). These data demonstrate that purine metabolic enzyme genes are new poor prognostic factors for NBs.

We further analyzed the human brain development (BrainSpan atlas) by the R2 data platform and revealed that there is an overall trend of a gradual decrease in purine enzyme gene expression from 8 weeks of age after fertilization to the prenatal period and that the purine enzyme gene expression levels are significantly lower at the postnatal stage than those at the prenatal period (Figure 2G, Figure S5A–D). We also downloaded a published single‐cell RNA‐seq data of adrenal gland development [17] and identified the sympathetic neuroblasts by examining the expression of specific markers, including peripherin (*PRPH*), stathmin 2 (*STMN2*), insulin gene enhancer binding protein 1 (*ISL1*), and tyrosine hydroxylase (*TH*). In the sympathetic neuroblasts, we found that *GART* gene expression varied along with the changes in *MYCN* gene expression under the development process, which provided valuable insight into the association of purine metabolic enzyme gene expression with the cell of origin of NB (Figure 2H, Figure S6A,B). Thus, our findings demonstrate that elevated expression of purine metabolic enzyme is not only associated with poor prognosis in NB patients but also inversely correlated with neurodevelopmental differentiation status. These data suggest that high expression of purine nucleotide metabolism enzymes is a new poor prognostic factor for NBs.

### 3.3 Targeting GART Restores NB Differentiation

**FIGURE 3:**
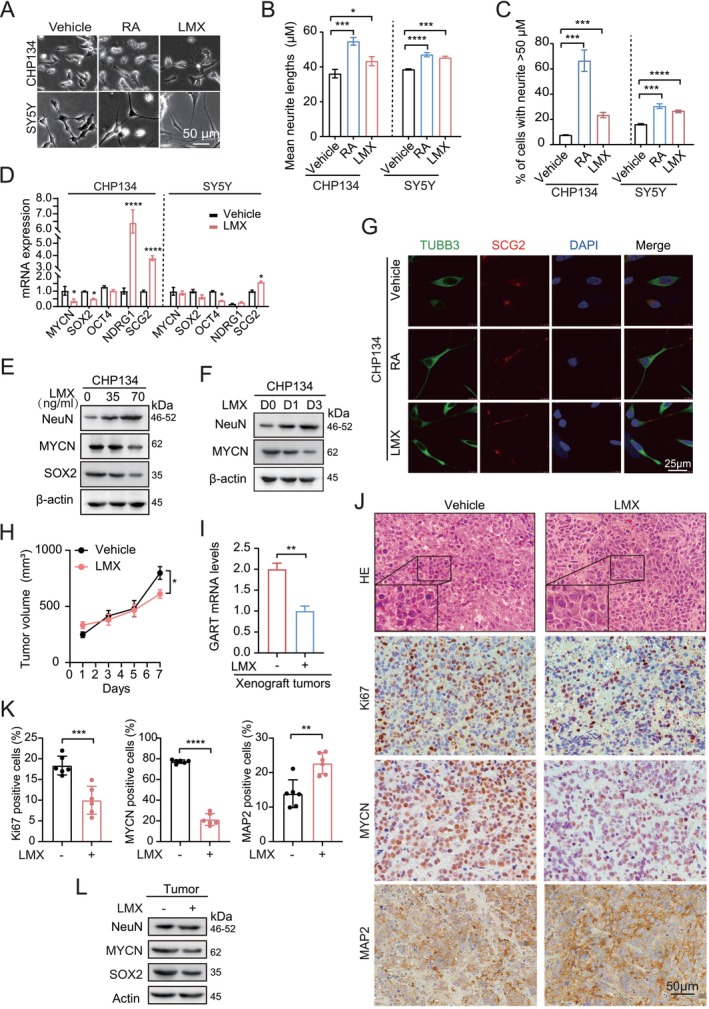
Targeting *GART* restores NB differentiation. (A) Bright‐field images of neurite outgrowth in CHP‐134 and SH‐SY5Y cells. Cells were treated with RA (3 μg/mL) or lometrexol (LMX, 5 ng/mL) for 3 days. Scale bars: 50 μm. (B) Mean neurite lengths in A. (C) The percentage of cells exhibiting one or more neurites (> 50 μm). Neurite length was quantified by Image J. (D) The mRNA expression of *MYCN, SOX2, OCT4, NDRG1*, and *SCG2* in A. (E) Western blot analysis of the effects of LMX on NeuN, MYCN, and SOX2 protein expression. CHP‐134 cells were treated with 0, 35, and 70 ng/mL LMX for 3 days, respectively. (F) Western blot analysis of MYCN and NeuN protein expression in CHP‐134 cells after 35 ng/mL LMX treatment for 0 day (D0), 1 day(D1), or 3 days (D3). (G) Immunofluorescence staining of TUBB3 (green), SCG2 (red), and DAPI (blue) in CHP‐134 cells. The treatment was the same as A. Scale bars, 25 μm. (H) Tumor growth curves of indicated CHP‐134 xenografts. When tumors reached a palpable size, the mice were treated with LMX (15 mg/kg) every 3 days by intraperitoneal injection two times. Tumor growth was monitored every other day. (I) qRT‐PCR analysis of the effects of LMX on GART expression in tumor cells from CHP‐134 xenografts. (J) Representative images of H&E and immunohistochemistry staining showed the proliferation index, Ki‐67, MYCN, and MAP2 expression in CHP‐134 xenograft tumors. Scale bars, 50 μm. (K) Quantification of cell numbers with a positive signal from J. (L) Western blot of SOX2, MYCN, and NeuN protein expression in CHP‐134 xenograft tumors. The original gel images of E, F, and L are presented in Figure S9. *p < 0.05, **p < 0.01, ***p < 0.001, ****p < 0.0001, by unpaired two‐tailed *t*‐test (B–D, H, I, and K).

To assess the function of purine metabolites on NB differentiation, we treated *MYCN*‐amplified (CHP‐134) and non‐amplified (SH‐SY5Y) cells with the selective GART inhibitor LMX for 3 days [18]. Using retinoic acid (RA) as a positive control, we observed that LMX (5 ng/mL) markedly promoted neurite outgrowth (Figure 3A,B) and augmented the proportion of cells with neurites > 50 μm in both cell lines (Figure 3C).

To further investigate the effects of LMX on NB cell differentiation, we assessed the expression of stemness‐associated genes and neuronal differentiation marker genes. As shown in Figure 3D, LMX downregulated stemness‐associated genes, *MYCN* and SRY‐Box transcription factor 2 (*SOX2*) while upregulating differentiation markers, N‐Myc downstream regulated 1 (*NDRG1*) and secretogranin II (*SCG2*) in *MYCN*‐amplified CHP‐134 cells. LMX downregulated the stemness‐associated gene POU class 5 homeobox 1 (*OCT4 or POU5F1*) but upregulated *SCG2* in non‐*MYCN*‐amplified SH‐SY5Y cells. These observations were validated by western blotting. LMX reduced the protein expression of MYCN and SOX2 but increased the neuron marker neuronal nuclear antigen (NeuN) protein expression in CHP‐134 cells (Figure 3E, Figure S7A). LMX also decreased MYCN protein expression in a time‐dependent manner (Figure 3F, Figure S7B). Additionally, LMX promoted SCG2 expression in neurites in CHP‐134 cells (Figure 3G). These data indicate that targeting GART promotes NB cell differentiation.

To evaluate the therapeutic potential of GART inhibition in vivo, we treated CHP‐134 xenografts with LMX (15 mg/kg) administered every 3 days for two cycles. LMX significantly reduced tumor growth (Figure 3H). LMX markedly decreased GART expression in tumors compared to the control (Figure 3I). We performed histological analysis and found that tumor cells in the LMX treated group exhibited increased cell size (Figure S7C). LMX downregulated the protein expression of MYCN and the proliferation marker Ki67 while upregulating the differentiation marker microtubule‐associated protein 2 (MAP2) (Figure 3J,K). LMX treatment also reduced the expression of stemness‐associated proteins SOX2 and increased neuron marker NeuN protein expression in xenograft tumors (Figure 3L, Figure S7D). These results collectively demonstrate that targeting GART with LMX restores differentiation and inhibits tumor progression in NBs.

### 3.4 Targeting GART Also Suppress Cell Proliferation, Colony Formation, and Migration

**FIGURE 4:**
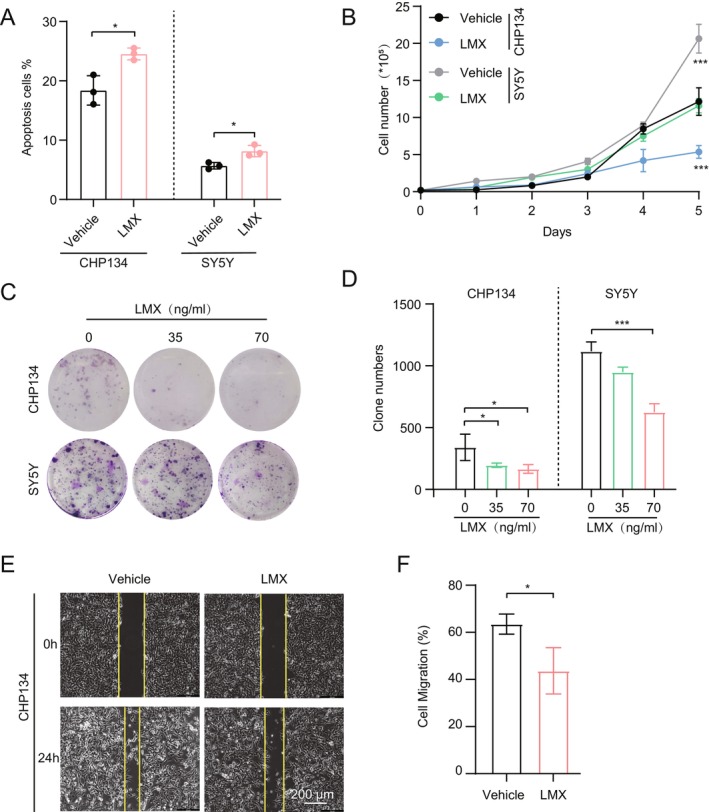
Targeting GART suppress cell proliferation, colony formation, and migration. (A) Apoptosis levels using flow cytometry. CHP‐134 and SH‐SY5Y cells were treated with or without 70 ng/mL LMX for 24 h. Early apoptosis cells were marked as (Annexin V‐APC)^+^/(7‐AAD)^−^, and the late apoptosis was marked as (Annexin V‐APC)^+^/(7‐AAD)^+^. This experiment included both early and late apoptosis. Each sample is processed with more than 1 × 10^5^ events. (B) Cell growth curve. CHP‐134 and SH‐SY5Y cells were treated with 70 ng/mL LMX. (C, D) Colony formation assay and quantification. CHP‐134 and SH‐SY5Y cells were treated with 0, 35, or 70 ng/mL LMX. (E, F) Wound healing assays and quantification. CHP‐134 cells were treated with 70 ng/mL lometrexol (LMX). At least three fields were captured for each sample; images were quantified by Image J. Scale bars, 200 μm. *p < 0.05, ***p < 0.001, by unpaired two‐tailed *t*‐test.

We further assessed the effects of GART inhibition on NB cell apoptosis. We performed flow cytometric analysis and revealed that LMX treatment significantly increased apoptosis rates in CHP‐134 and SY5Y cells compared to controls (*p* < 0.05) (Figure 4A, Figure S8). LMX treatment markedly reduced NB cell proliferation (Figure 4B). Colony formation assays demonstrated dose‐dependent inhibition of clonogenic potential in CHP‐134 and SH‐SY5Y cells (Figure 4C,D). Wound healing/transwell assays showed impaired migratory capacity following LMX exposure (Figure 4E,F). These results indicate the dual effects of GART inhibition, promoting differentiation while suppressing proliferation, survival, and migration.

## 4 Discussion

Patients with *MYCN*‐amplified NBs exhibit an exceptionally poor clinical prognosis, highlighting the critical demand for innovative therapeutic interventions. Our findings reveal that MYCN orchestrates purine nucleotide metabolism through transcriptional regulation of key purine biosynthetic enzymes. Importantly, pharmacological inhibition of GART using LMX not only induces NB differentiation but also significantly attenuates tumor cell proliferation, offering a compelling dual‐targeting approach for high‐risk NB treatment.

MYCN is a well‐characterized oncogenic driver that reprograms metabolic pathways in NBs. Previous studies have established that MYCN augments glutamine uptake and its subsequent conversion to α‐ketoglutarate to sustain tricarboxylic acid cycle activity [19, 20]. In this study, we identified that MYCN significantly upregulated key enzymes and metabolites associated with the purine synthesis pathway. Notably, compared to their non‐amplified counterparts, *MYCN*‐amplified NB cells exhibited significantly elevated levels of purine metabolites, including AICAR, IMP, and GMP, which implicate enhanced purine biosynthesis as a metabolic dependency for sustaining rapid proliferation in MYCN‐driven tumors [8]. *MYCN*‐amplified NBs coordinate carbon metabolism and purine biosynthesis through transcriptional upregulation of MYCN‐targeted metabolic genes, such as MTHFD2 and PAICS [8]. Our analysis further revealed that elevated expression of purine nucleotide metabolism enzymes (PRPS1, PPAT, PAICS, and PFAS) correlates with unfavorable clinical outcomes in NB patients. Among these, glycinamide ribonucleotide transformylase (GART) emerged as a pivotal regulator. As a key folate‐dependent enzyme in the de novo purine synthesis pathway, GART facilitates nucleotide production essential for DNA replication [21] and is frequently overexpressed in aggressive malignancies, including hepatocellular carcinoma [22], glioma [23], and colorectal cancer [24]. Importantly, our findings position GART as a potential prognostic biomarker and therapeutic target in high‐risk NBs.

LMX, a potent and selective glycinamide ribonucleotide transformylase (GART) inhibitor currently under clinical evaluation [18, 25], effectively disrupts de novo purine biosynthesis without inducing DNA strand breaks [26]. Our comprehensive in vitro and in vivo analyses demonstrate that LMX significantly suppresses NB proliferation, reduces tumor cell size, and alters cellular morphology. Furthermore, LMX treatment modulated tumor volume in preclinical models while concurrently promoting tumor cell differentiation, suggesting its potential as a synergistic adjunct to conventional chemotherapy. LMX‐mediated inhibition of purine metabolism perturbs cellular proliferation and triggers apoptotic pathways, consistent with its reported induction of neural tube defects in murine models [27]. Additionally, LMX attenuates serine hydroxymethyltransferase (SHMT) activity, thereby depleting the primary source of one‐carbon units essential for nucleotide biosynthesis [28]. These dual mechanisms of action—direct GART inhibition and secondary SHMT suppression—underscore LMX's unique therapeutic potential for targeting metabolic vulnerabilities in high‐risk NBs.

## 5 Conclusions

Collectively, our findings establish MYCN‐mediated purine metabolic dysregulation as a therapeutic vulnerability in NB. The differentiation‐inducing effects of LMX, demonstrated across preclinical models and supported by bioinformatic analyses, reveal its potential as a first‐in‐class differentiation therapy for high‐risk disease. This targeted metabolic intervention may complement existing regimens to improve both treatment efficacy and quality of survival for NB patients.

## Author Contributions

**Yufeng Jiang:** investigation (equal), methodology (equal), software (lead), visualization (lead), writing – original draft (lead). **Hui Xiao:** software (equal), validation (equal), visualization (equal). **Yi Yang:** software (equal), validation (equal). **Guoyu Chen:** software (equal). **Yingwen Zhang:** methodology (equal), resources (equal). **Xiaoyu Wu:** software (equal). **Qi Zhang:** investigation (equal), validation (equal). **Qingyi Huang:** investigation (equal), validation (equal). **Hongxiang Gao:** software (equal), validation (equal). **Yali Han:** software (equal), validation (equal). **Anan Zhang:** software (equal), validation (equal). **Dapeng Jiang:** validation (equal). **Liang Zheng:** conceptualization (equal), methodology (equal), writing – review and editing (equal). **Yanxin Li:** conceptualization (equal), funding acquisition (equal), writing – review and editing (equal). **Yijin Gao:** methodology (equal), supervision (equal), writing – review and editing (equal).

## Ethics Statement

The study was approved and supervised by the SCMC Ethics Committee (SCMCIRB‐K2014050), according to the Declaration of Helsinki. All subjects provided written consent for the banking of tissue and future research use of the samples, following the regulations of the institutional review board of SCMC.

## Consent

The authors have nothing to report.

## Conflicts of Interest

The authors declare no conflicts of interest.

## Supporting information


**Figure S1:** *MYCN* knockdown reduces the expression of purine metabolism enzyme *FGAM*.
**Figure S2:** *MYCN* regulation of purine metabolic enzyme gene expression and correlation of purine metabolic enzyme gene expression with *MYCN* amplification, risk, progression, and stages.
**Figure S3:** High expression of purine metabolism enzyme genes is related to a poor prognosis in NBs.
**Figure S4:** Purine metabolic enzyme genes are independent risk factors of NBs.
**Figure S5:** Purine enzyme gene expression levels are significantly lower at the postnatal stage than at the prenatal period.
**Figure S6:** The expression levels of purine metabolizing enzyme genes changes with embryonic development.
**Figure S7:** Targeting GART induce NB differentiation.
**Figure S8:** Lometrexol treatment significantly increased cell apoptosis.
**Figure S9:** Full scans of western data. Rectangles delimit cropped areas used in the indicated figures.
**Table S1:** Primer list.

## Data Availability

All data needed to evaluate the conclusions in the paper are present in the paper and/or the Supporting Information S1. Additional data related to this paper may be requested from the authors.
