# Supplementary material for: Inhibition of Purine Metabolism Promotes the Differentiation of Neuroblastoma Driven by MYCN
Source: Cancer Med. 2025 May 12;14(9):e70953. doi: 10.1002/cam4.70953 (PMC12067188; doi:10.1002/cam4.70953)
Supplement: Supplementary file 1 — Figure S1: MYCN knockdown reduces the expression of purine metabolism enzyme FGAM. Figure S2: MYCN regulation of purine metabolic enzyme gene expression and correlation of purine metabolic enzyme gene expression with MYCN amplification, risk, progression, and stages. Figure S3: High expression of purine metabolism enzyme genes is related to a poor prognosis in NBs. Figure S4: Purine metabolic enzyme genes are independent risk factors of NBs. Figure S5: Purine enzyme gene expression levels are significantly lower at the postnatal stage than at the prenatal period. Figure S6: The expression levels of purine metabolizing enzyme genes changes with embryonic development. Figure S7: Targeting GART induce NB differentiation. Figure S8: Lometrexol treatment significantly increased cell apoptosis. Figure S9: Full scans of western data. Rectangles delimit cropped areas used in the indicated figures. Table S1: Primer list. [file CAM4-14-e70953-s001.docx]

**Supporting Information**

**Inhibition of purine metabolism promotes the differentiation of the neuroblastoma driven by *MYCN***

Yufeng Jiang *et al.*

*Corresponding author: Yanxin Li, E-mails: liyanxin@scmc.com.cn; Liang Zheng, E-mails: [lzheng@shsmu.edu.cn](mailto:lzheng@shsmu.edu.cn); or Yijin Gao, E-mails: gaoyijin@scmc.com.cn

**This PDF file includes:**

Fig. S1-S9

Table S

**Figure S1. *MYCN* knockdown reduces the expression of purine metabolism enzyme *FGAM*.**

1. *MYCN* knockdown decreases the expression of purine metabolism enzyme *FGAM* in *MYCN*-amplified SH-SK-N-BE(2) cells. sgCK, a control sgRNA. sg*MYCN*, a *MYCN* sgRNA.
2. Relative levels of FGAM in *MYCN*-amplified CHP-134 cells with or without *MYCN* sgRNA.

**Figure S2. *MYCN* Regulation of Purine Metabolic Enzyme Gene Expression and Correlation of Purine Metabolic Enzyme Gene Expression with *MYCN* Amplification, Risk, Progression, and Stages**

1. qRT‑PCR analysis of the effects of *MYCN* knockout or overexpression (OV) on purine metabolic enzyme gene expression in NB cells.

B-D) Correlation of purine metabolic enzyme gene expression with *MYCN* status (B), risk (C), and progression (D) in the SEQC cohort (GSE62564).

E-L) Expression levels of purine metabolic enzyme genes in different NB stages in the SEQC cohort (GSE62564).

* *P* < 0.05, *** *P* < 0.001, **** *P* < 0.0001, by unpaired two-tailed *t*-test.

**Figure S3. High Expression of Purine Metabolism Enzyme Genes Is Related to A Poor Prognosis in NBs**

A) Kaplan-Meier analysis of overall survival of NB patients with high or low gene expression of *PRPS1*, *PPAT*, *PFAS*, and *PAICS* in the SEQC cohort (GSE62564). The best cut-point was employed as the cutoff.

B) Kaplan-Meier analysis of event-free survival of NB patients with high or low gene expression of *PRPS1*, *PPAT*, *PFAS*, and *PAICS* in the SEQC cohort (GSE62564). The best cut-point was employed as the cutoff. **** *P* < 0.0001, by log-rank test.

**Figure S4. Purine Metabolic Enzyme Genes Are Independent Risk Factors of NBs.**

Multivariate Cox regression analysis of the prognostic ability of *PRPS1* (**A**), *PPAT* (**B**), *PFAS* (**C**), *PAICS* (**D**), *ADSL* (**E**), *ATIC* (**F**), or *GMPS* (**G**) expression in the SEQC cohort (GSE62564).


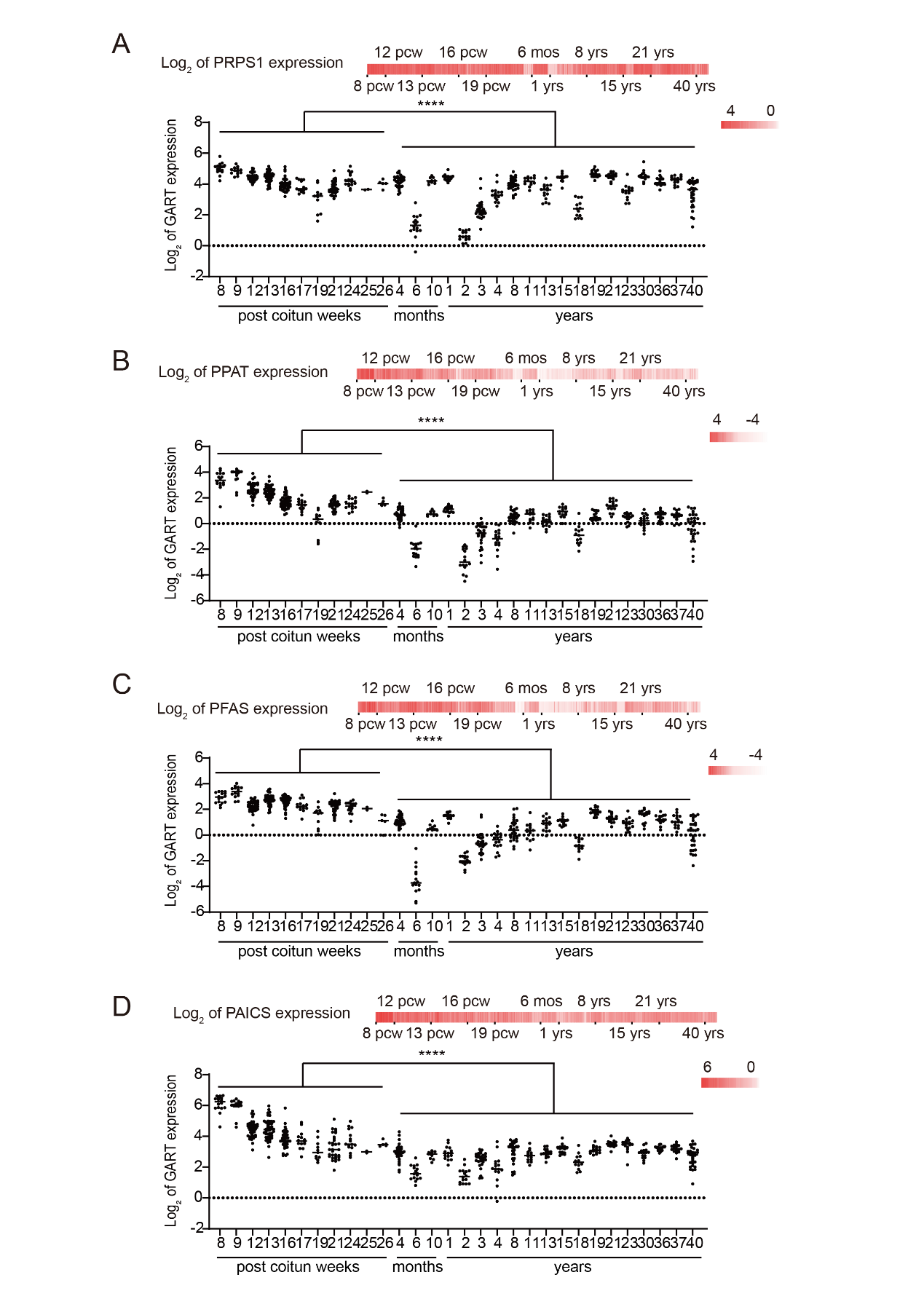


**Figure S5. Purine Enzyme Gene Expression Levels Are Significantly Lower at the Postnatal Stage than at the Prenatal Period.**

Barplot showing the log2 transformed fold-change in *PRPS1* (**A**), *PPAT* (**B**), *PFAS* (**C**), and *PAICS* (**D**) expression during normal human brain development (upper panel) with grouped scatter plots (lower panel). pcw, post coitum weeks indicates post fertilization weeks. m, months indicates months. y, years indicates ages. Data from BrainSpan expression profiles (n=524) in the R2 data platform (r2.amc.nl). **** *P* < 0.0001, by unpaired two-tailed *t*-test.

**
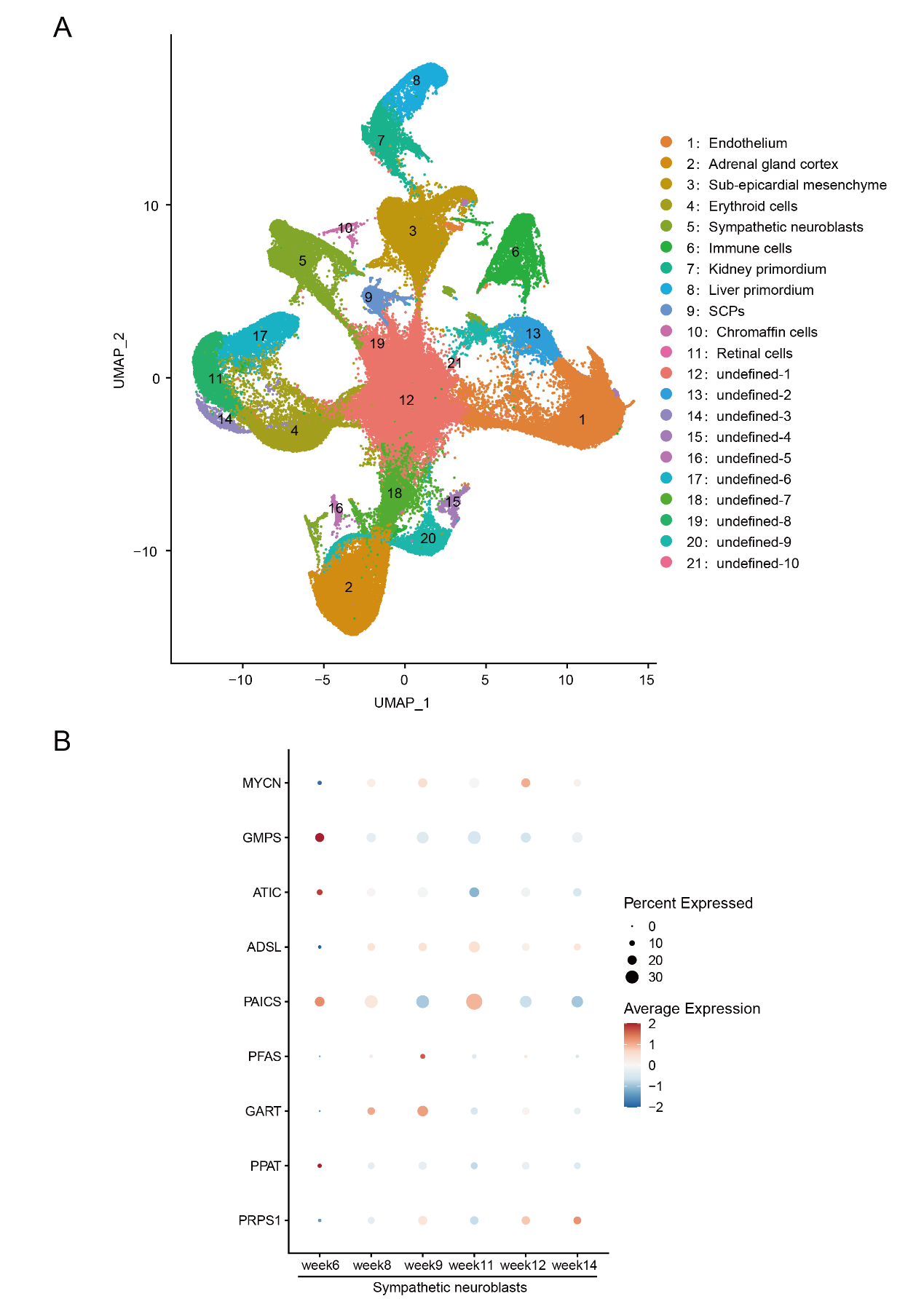
**

**Figure S6. The Expression Levels of Purine Metabolizing Enzyme Genes Changes with Embryonic Development**

A) A UMAP embedding of the 146275 cells, color-coded by tissue type for each cluster.

B) Dot plot of *MYCN* and purine metabolic genes in sympathetic neuroblasts with different embryonic weeks.

**
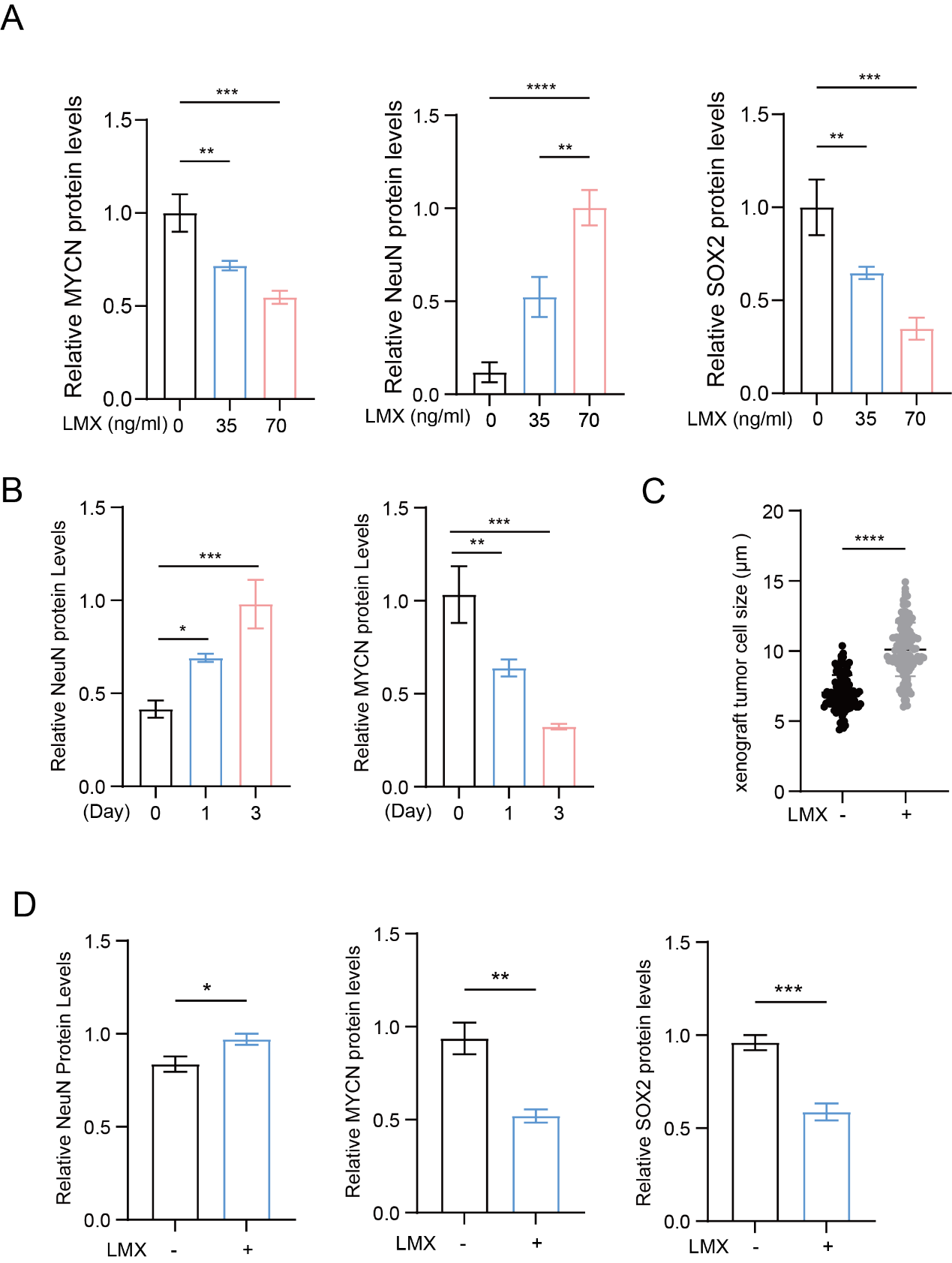
**

**Figure S7. Targeting GART Induce NB Differentiation.**

A) Quantification of protein bands in Figure 3E using ImageJ.

B) Quantification of protein bands in Figure 3F using ImageJ.

C) Maximum diameter of tumor cells from CHP-134 xenografts in the control group and after lometrexol treatment.
D) Quantification of protein bands in Figure 3L using ImageJ.

* *P* < 0.05, *** *P* < 0.001, **** *P* < 0.0001, by unpaired two-tailed *t*-test (A, B, C and D)

**
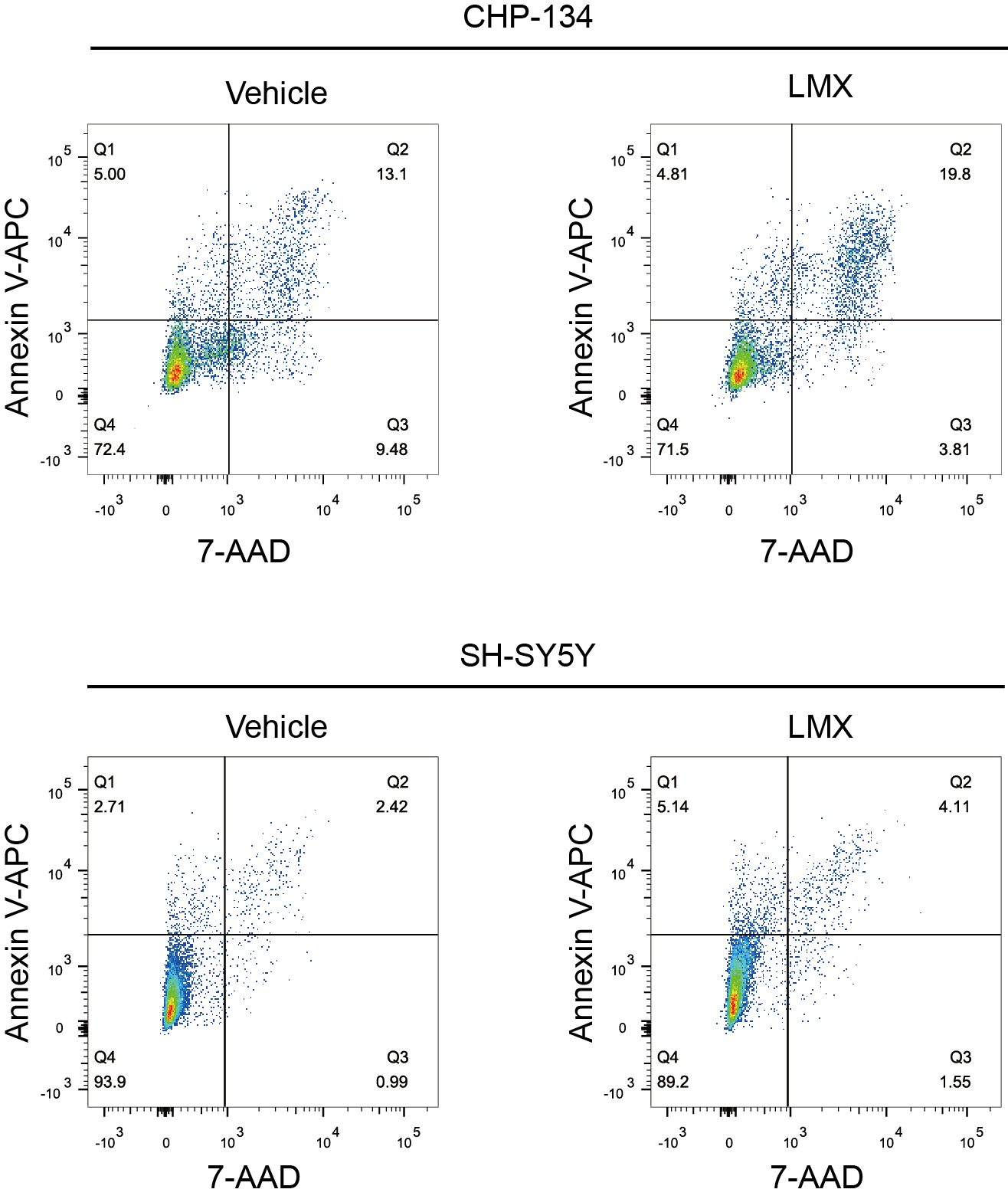
**

**Figure S8. Lometrexol treatment significantly increased cell apoptosis**

Early apoptosis cells were marked as (Annexin V-APC)^+^/(7-AAD)^-^, which was shown as Q1, and the late apoptosis was marked as (Annexin V-APC)^+^/(7-AAD)^+^, which was showed as Q2. This experiment included both early and late apoptosis. Each sample is processed with more than 1×10^5^ events.

**
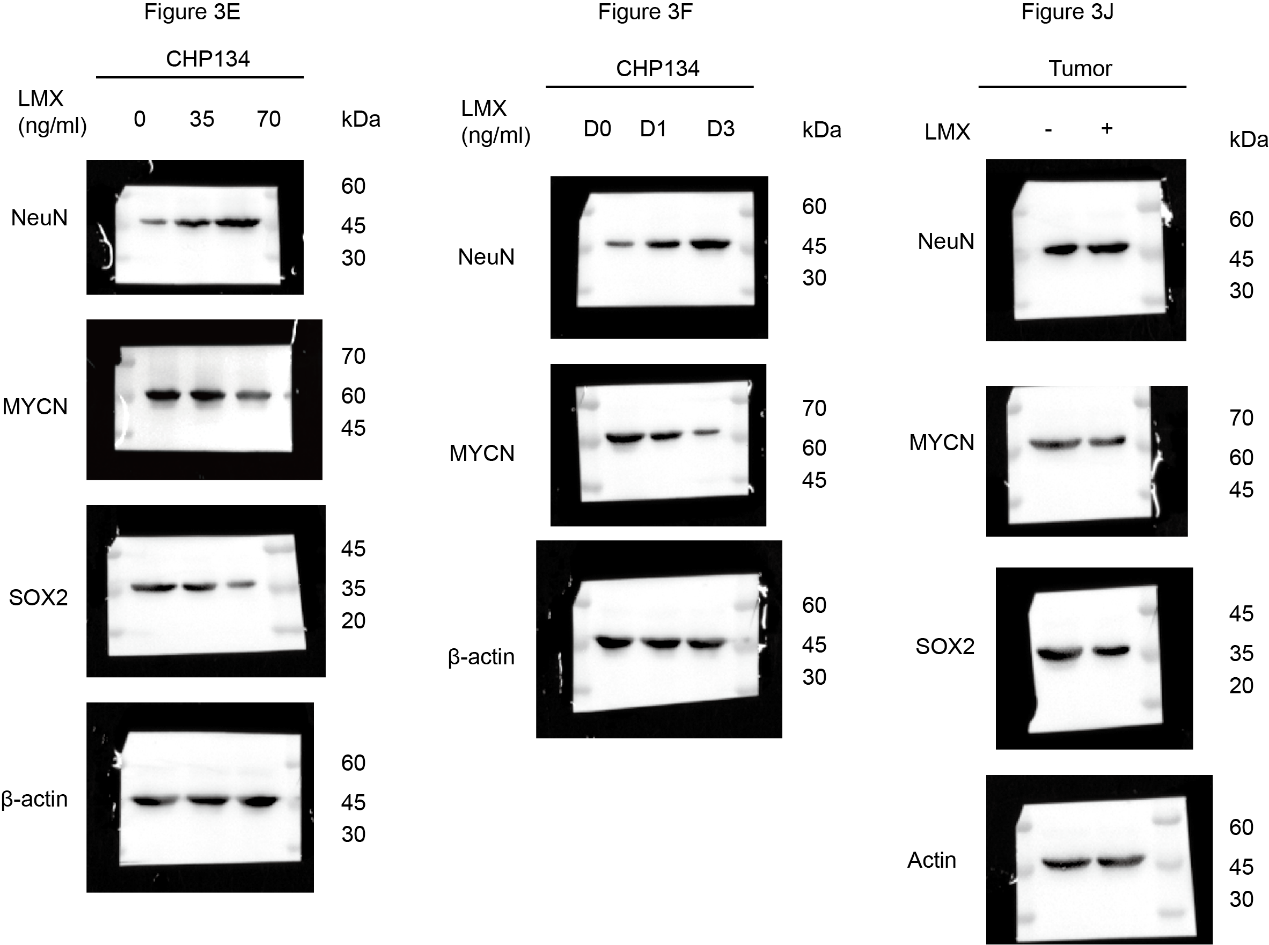
**

**Figure S9. Full scans of Western data. Rectangles delimit cropped areas used in the indicated figures.**

**Table S1 Primer list.**

| Gene | F(5’-3’) | R(3’-5’) | |
| --- | --- | --- | --- |
| *OCT4* | TGTACTCCTCGGTCCCTTTC | TCCAGGTTTTCTTTCCCTAGC | |
| *SOX2* | GCTAGTCTCCAAGCGACGAA | GCAAGAAGCCTCTCCTTGAA |  |
| *MYCN* | CAGTCTGGACACTGGCTGAA | CTCGCTGATTAGGCTCCAAC |  |
| *NDRG1* | CTGAAATGCTTCCTGGAGTCCT | TCCACCATCTCAGGGTTGTTTAG |  |
| *SCG2*  *PRPS1*  *PPAT*  *PFAS*  *PAICS*  *ADSL*  *ATIC*  *GMPS* | GAAGCCGAATGGATCAGTGGA  ATCTTCTCCGGTCCTGCTATT  AATTGTCAGCCCTTCGTTGTT  ATGTGGCTGTTTGGTTGCC  ACAAACAGTCTTATCGGGACCT  TTACGACATGATGTGATGGCTC  GCAGCCAAAAACCACGCTC  AGGAACAAGGATTCCGTGCTA | TGGTCTAAGTCAGCCTCTGAG  TGGTGACTACTACTGCCTCAAA  CCTTAATCGAGCAGCATTTACCA  CACACTGACACGATGTTGGT  CTGCAACCCACTCAAAGTTTTTC  TCCAACATAGCAAGAAGTAGCAC  CTGGCGTCTAGTCTCCAAGGA  GAAGGCCCCTGAATAATGAACA |  |
| *ACTB* | TGACGTGGACATCCGCAAAG | CTGGAAGGTGGACAGCGAGG |  |
